# Why we sometimes punish the innocent: The role of group entitativity in collective punishment

**DOI:** 10.1371/journal.pone.0196852

**Published:** 2018-05-03

**Authors:** Andrea Pereira, Jan-Willem van Prooijen

**Affiliations:** 1 Department of Psychology, New York University, New York, NY, United States of America; 2 Social and Organizational Psychology Department, Leiden University, Leiden, The Netherlands; 3 Department of Experimental and Applied Psychology, VU Amsterdam, Amsterdam, The Netherlands; 4 Netherlands Institute for the Study of Crime and Law Enforcement, Amsterdam, The Netherlands; Mälardalen University, SWEDEN

## Abstract

Because punishments are expected to give offenders what they deserve proportionally to the severity of their offenses, the punishment of an entire group because of the misdeed of a few of its members is generally considered as unfair. Group entitativity might increase support for such collective punishment, because members of highly entitative groups are perceived as more similar and interchangeable. We designed three experiments comparing support for third-party collective punishment of low versus high entitative groups. As comparison base-rate, we included conditions in which participants punish an individual wrongdoer (Experiments 1 & 2). Results show that although support for individual punishment is higher than support for collective punishment, this difference was reduced (Experiment 1) or absent (Experiment 2) when the group was highly entitative. Experiment 3 replicated the increasing effect of group entitativity on support for collective punishment. We conclude that group entitativity increases the likelihood of an entire group being treated as a single unit, facilitating collective punishment when a few group members commit an offense.

## Introduction

During the finals of the national Dutch cup in April 2014, a few Ajax supporters interrupted the game by throwing fireworks on the field, and they inflicted severe damage to several areas of the soccer stadium. Although the damage was caused by only a handful of hooligans, the entire club was subsequently fined with €70’000, and the decision was made to ban all supporters—including the innocent majority—from attending the future games between these two teams for the following three years. Clearly, an entire group—the soccer club Ajax and its supporters—was punished for the behavior of only a few of its members. This example illustrates a situation in which only a few group members perpetrate a wrongdoing, leading external authorities to inflict a punishment upon all members of the group. Whereas many people would consider the punishment of innocent group members, because of the misdeeds of others, blatantly unfair, this example reveals that such collective punishment actually emerges frequently in everyday social life [1,2], as examples abound in school classrooms, sport teams, the military, traditional societies [3], or, at the international level, in the cases of embargos and wars, and many other social situations.

What might generate support for punishment that also targets people who carry no guilt except for sharing a group membership with the offender? How does such collective punishment compare with individual punishment? In spite of the importance and relevance of such questions, very little research offers insights into the factors that drive the willingness to inflict collective punishments. In the present paper, we argue that although people generally support the punishment of an individual wrongdoer more strongly than that of a group, this difference can be reduced when the group is not perceived as a collection of separate individuals, but as one coherent entity. Stated otherwise, highly entitative groups, such as soccer fans are likely to be perceived, should more easily be the targets of collective punishment than low entitative groups.

### Punishing the innocent

People experience an impulse to punish others, i.e. to intentionally apply a negative sanction upon someone [4] for having committed an action considered as wrong [5]. The literature suggests that people’s punitive intentions are mostly guided by just deserts motives, rather than by utilitarian punishment goals [5–8]: People want to see culprits get what they deserve as a function of the morality of their actions. Such retributive motives suggest that punishing innocent people should be perceived as immoral and unfair. In spite of that, punishments often are inflicted upon people for a wrongdoing that they did not commit: Collective punishments entail situations in which an entire group is punished for a wrongdoing perpetrated only by a subset of its group members [9]. Support for such treatments has been shown to be shaped by the group’s political organization [9], collective responsibility [10], as well as by perceptions of value-violations from the offender group [11].

Hence, on the one hand people punish in order to give offenders what they deserve, but on the other hand they also sometimes punish innocent individuals for the sole reason that they share a group membership with an actual offender. Considering these two seemingly irreconcilable observations, one might wonder what drives people’s impulse to punish groups after a wrongdoing perpetrated by a few group members. The present research proposes that people are more willing to inflict third-party collective punishments when people perceive the group as a single entity rather than as a collection of distinct individuals. In order to address this question, we examine the effect of group entitativity on support for collective punishment.

### Entitativity

Social groups can differ on the extent to which they are perceived as one coherent unit composed of well-connected group members, or in contrast as a cluster of individuals loosely associated. Scholars use the terms *entitativity* to refer to this property of groups [12]. Highly entitative group members are perceived as strongly interacting with each other, ascribing more importance to their group, having common outcomes and goals for the group and being similar to each other [13]. In other words, entitativity is linked to a higher cohesiveness and “groupness”.

Higher levels of entitativity appear to increase expectations of internal cohesion and consistence in groups [14]. For example, the overattribution bias [15] has been shown to emerge when the target is a high entitative group, but not a low entitative group [16]. In addition, entitativity facilitates the transmission of attributes from one group member to the rest of the group [17]. This suggests that members of highly entitative groups might be perceived as relatively interchangeable, and their future behavior might be expected to be more consistent and predictable as compared to that of members of lowly entitative groups.

If entitativity increases perceptions of consistency, it might also affect the way outsiders react following a wrongdoing committed by some of the members of a particular group. Given that highly entitative groups are perceived as a cohesive unit, individual offenders are likely to reflect upon their entire group. Consistent with this, higher levels of entitativity increase perceptions of collective responsibility for group actions [18] and for wrongdoings [19,20]. Additionally, studies have shown that group entitativity also increases willingness to inflict different types of punishment [21–25], to the extent that the target group is not in a position to retaliate [26].

Whereas the above studies establish a link between group entitativity and direct retaliation, none of these previous studies has specifically focused on third-party collective punishment, i.e. the punishment of an entire group for the misdeed of a few group members by an external agent [9,10]. Indeed, goup punishment judgments examined in that earlier work were mostly second-party judgments, such as *retaliatory* collective punishments by the victim following a rejection [24] or a provocation [26], and vicarious punishments (or group-based retaliation)—i.e. the *displacement* of a punishment from the original outgroup offender to a fellow group member [21,22]. By focusing on third-party collective punishment, the present research is designed to meaningfully extend these earlier studies. First, the retaliatory responses that were observed in previous studies constituted a response to harm that participants directly experienced (for example, having to drink a very sour drink that was picked by another group; see Study 1 in [24]). In our work, we examine people’s punitive responses as an external observer. Whereas it is well known that third parties engage in retributive punishment in response to norm violations harming unrelated others [5], and are even willing to pay in order to punish offenders [27,28], it is less clear that independent third parties would punish innocent people simply for sharing a group membership with the offender, and that this punishment would be affected by entitativity perceptions.

Second, and relatedly, by focusing on the willingness to punish out of a desire for revenge, previous studies typically examined the effects of strong, anger-based emotions [25,29]. Such emotions are likely to lead to an overheated response, and to be biased by factors irrelevant to this actual misdeed, such as the group’s entitativity. Indeed, revenge and third party punishment differ crucially in their underlying goals: Whereas revenge is focused on making offenders suffer, and is even associated with a willingness to deny the offender fair procedures, the just deserts perspective that is typically associated with third party punishment is predominantly focused on restoring the values that were violated, hence re-establishing a sense of justice [30]. Third-party punishment is also widely considered to be more legitimate than second-party revenge [8]. One could therefore expect a bias as a function of group entitativity to be less likely to occur when the judgment is made by a third party, because they are in an a priori neutral position *vis-à-vis* the actors.

The present research investigates the effect of group entitativity in situations in which a third party observes a wrongdoing, and addresses the specific question to what extent the observer is willing to punish the entire group to which the wrongdoer belongs, hence punishing innocent group members. As members of social groups and institutions rarely act all together as one, it is important to understand the judgments likely to occur in situations in which they have to collectively suffer the consequences of some of their individuals’ misbehavior. It seems, then, that justice-related judgments directed at high entitative groups are different from those directed at low entitative groups, in the sense that collective punishment is considered more acceptable when the group is high on entitativity.

### Overview and hypotheses

The present paper describes three experiments that investigate the question whether group entitativity influences the collective punishment that third parties assign. In all experiments, we presented participants with a wrongdoing and measured their support for a punishment in reaction to this wrongdoing. In order to have a comparison base-rate, we compared collective punishment with individual punishment in Experiments 1 and 2. Although the wrongdoing was always committed by a single or a couple of individual(s), the target of punishment was manipulated: either the actual wrongdoer (individual conditions) or the group to which the wrongdoer belonged (collective conditions). In addition, the group’s level of entitativity was manipulated (low vs. high) in all experiments. Experiments 1 and 2 included an individual condition, and Experiment 1 included an additional control condition where no offense was committed. As a result, Experiment 1 consisted of a 4-cell design, Experiment 2 of a 3-cell design, and Experiment 3 of a 2-cell design, the latter focusing solely on the critical group conditions. Furthermore, Experiments 1 and 3 were vignette experiments conducted online, whereas Experiment 2 was a lab study in which participants witnessed a confederate commit the wrongdoing. We expected support for punishment to be lowest in the control condition, and highest in the individual wrongdoer condition. Moreover, we expected support for collective punishment to be higher in the high entitativity condition as compared to the low entitativity condition. In other words, people should be more supportive of the punishment of an individual wrongdoer than that of a group, but this difference should be reduced when the group’s entitativity is high rather than low.

## Experiment 1

### Method

#### Participants and design

In Experiment 1, we recruited 203 participants on Amazon Mechanical Turk. A sensitivity power analysis confirmed that for the crucial contrast testing our hypothesis (see results section) this sample yields 80% power for a small to medium effect size (*f*^*2*^ = .04). Their ages ranged from 18 to 68 (*M* = 34.79, *SD* = 10.63), and 48 percent of them were female. They were randomly assigned to one out of four conditions in a four-cell design: control, individual, low entitativity group, and high entitativity group. This experiment, as well as Experiments 2 and 3, was conducted in accordance with the ethical standards and with explicit approval of the Scientific and Ethical Review Board of the Faculty of Behavioural and Movement Sciences of the VU Amsterdam. Consent was explicitly given by each participant.

#### Procedure

Following the procedure used in previous research [9,10], participants read about teenagers taking part in a summer camp and answer questions regarding those teenagers and an incident that supposedly happened during that camp.

#### Independent variable

**Fig 1:**
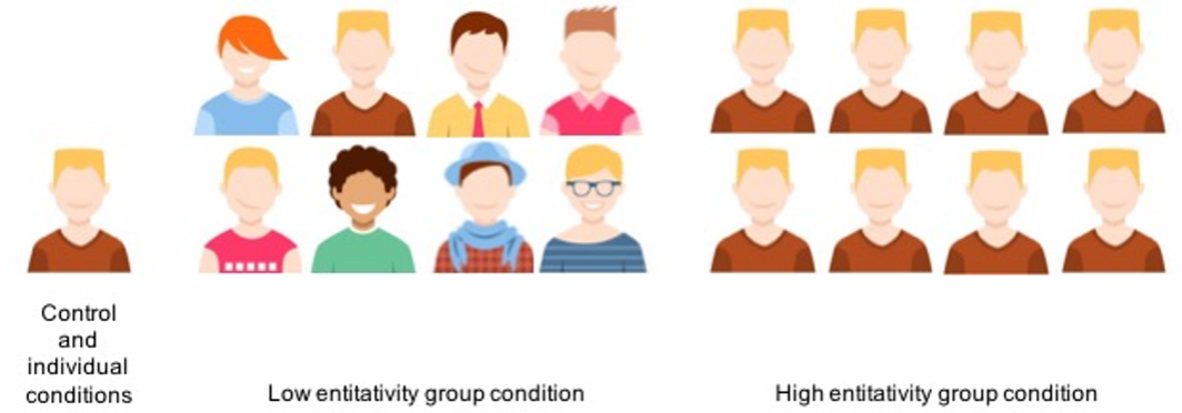
Pictures used along the descriptions of the punishment targets in every condition (Experiment 1).

We manipulated the target of the punishment: In the *individual and control* conditions, the survey mentions one teenager that joins a summer camp composed of teenagers who did not know each other. In the group conditions, the survey mentions a group of teenagers who go on a summer camp. We manipulated group entitativity based on previous studies that have successfully measured and manipulated this dimension [10,13]. The *low entitativity group* condition reports that the summer camp is composed of teenagers who did not know each other and were assembled randomly by the camp organizers. The *high entitativity group* condition describes a group composed of teenagers who were friends and members of the same sports team and gathered together in the same camp out of affinity. A picture of the individual or the group in question was presented along with each description (cf. Fig 1).

After describing the target of the punishment, the survey went on to describe the teenagers’ behavior that the survey focused on. It said that people from a nearby village complained about the noise related to the summer camp and tried to have it closed. The *control* condition said that a few days later, the teenager went to the nearby village and had a conversation with the villagers about the camp's nuisances. The *individual* condition said that a few days later, the teenager went to the village and sprayed graffiti on facades, broke windows, insulted a resident and stole items from the local supermarket. The *groups* conditions described the same acts of vandalism, but those were committed by two members of the group that could not be identified.

#### Manipulation checks

Right after the description of the target of punishment, but before the description of the wrongdoing, we assessed perceptions of *entitativity* using the items from previous research on group entitativity [20,31]. On a 7-points scale (1 = *Not at all*, 7 = *Absolutely*), participants indicated to what extent they thought these teenagers: *were similar to each other*, *had similar goals*, *interacted with each other* and *thought this group was important to them* (α = .83). In the control and individual conditions, we used similar items rephrased to focus on the individual teenager as filler items (e.g., *he/she is similar to the other teenagers*).

#### Dependent variable

After the wrongdoing’s description, participants indicated on a 7-points scale (1 = *Not at all*, 7 = *Absolutely*) the extent to which some forms of punishment should be inflicted upon the target (i.e., the individual or the group, depending on condition): *publicly apologize to the victims and to the village authorities*, *complete community service hours in the village*, *pay a compensation to the village*, *be sent home before the end of the summer camp*, *do additional chores in the camp*, *be deprived from enjoyable activities*, *be awaken earlier in the morning* (α = .92).

Besides the measures reported in this paper, we measured a range of other constructs in all three experiments which are reported, and analyzed, in the Online Supplementary Materials (see S1 File).

### Results

Means and Standard Deviations for all studies are displayed in Table 1.

**Table 1:** Means and Standard Deviations for entitativity perceptions and support for punishment as a function of the experimentl conditions (Experiments 1, 2 & 3).

	Control	Individual target	Low entitativity group target	High entitativity group target
Experiment 1				
Entitativity	–	–	4.71^a^ (0.79)	6.15^b^ (0.72)
Punishment	2.23^a^ (1.40)	5.57^b^ (0.88)	4.20^c^ (1.50)	4.78^d^ (1.64)
Experiment 2				
Entitativity	–	–	4.08^a^ (1.24)	5.11^b^ (0.90)
Punishment	–	2.52^a^ (1.20)	1.71^b^ (0.97)	2.46^a^ (1.39)
Experiment 3				
Entitativity	–	–	4.04^a^ (1.34)	6.09^b^ (0.93)
Punishment	–	–	4.33^a^ (1.36)	4.78^b^ (1.10)

Means not sharing subscripts differ at least at *p* < .05.

#### Manipulation checks

A t-test revealed that, as expected, the group was perceived as less entitative in the low entitativity condition than in the in high entitativity condition, *t*(106) = -9.70, *p* < .001. This finding indicates that participants distinguished between the groups high versus low on entitativity as intended.

#### Dependent variable

We performed planned orthogonal contrasts analyses using regression analyses. We created three orthogonal contrasts testing whether support for punishment was significantly lower in the control condition as compared all three conditions where an offense was committed (C1: -3, 1, 1, 1), higher in the individual condition as compared to both group conditions (C2: 0, 2, -1, -1), and lower in the low entitativity group condition as compared to the high entitativity group condition (C3: 0, 0, -1, 1), the latter being the critical test of our hypothesis. Results show that all three contrasts were significant. The target was punished less severely in the control condition as compared to all other conditions, C1: *B* = .65, CI_95%_ [.54; .77], β = .62, *t*(199) = 11.60, *p* < .001, *d* = .35. Support for the individual punishment was also higher than support for the group conditions, C2: *B* = .36, CI_95%_ [.20; .52], β = .23, *t*(199) = 4.32, *p* < .001, *d* = .19. Finally, support for collective punishment was higher when the group’s entitativity was high rather than low, C3: *B* = .29, CI_95%_ [.02; .56], β = .11, *t*(199) = 2.13, *p* = .034, *d* = .16. This latter finding supports our hypothesis. Given that gender and age interacted with some of the contrasts to predict support for collective punishment (gender interacted with C3, *B* = -.31, *p* = .030; and age interacted marginally with C1, *B* = .01, *p* = .093), we conducted the same analyses controlling for age and gender; the results were significant and strictly identical (see S1 File for details).

### Discussion

Results from Experiment 1 supported our reasoning. First, and not surprisingly, support for punishment was lowest when no wrongdoing had been committed (control condition) as compared to all other conditions. Second, support for punishment was highest when the target was an individual wrongdoer as compared to when the target was a group. Finally, and in line with our predictions, support for collective punishment was higher when the group was high rather than low on entitativity. One weakness of the present experiment is that while only one offender committed the misdeed in the individual condition, they were two offenders in the group conditions (both low and high entitativity). Although this cannot account for the observed difference in support for collective punishment between the low and the high entitativity conditions, one could argue that the likelihood of collective punishment is overall increased because multiple members of the group are guilty of the offense rather than only one. In the second experiment, we wanted to replicate these findings while keeping one unique offender in all conditions. In order to assess whether these findings would generalize to situations where people directly witness an offense, Experiment 2 was conducted in the lab by confronting participants with an actual wrongdoing perpetrated by a confederate.

## Experiment 2

### Method

#### Participants and design

In Experiment 2, we required a minimum of 20 participants per cell a priori, taking into consideration the work load related to a lab study with three confederates, and testing each participant separately. Participants were 62 students from a large Dutch university. Although this sample is small for our purposes, note that for the crucial contrast testing our hypothesis (see results section) this sample still yields 80% power for an effect size close to medium (*f*^*2*^ = .13). Their ages ranged from 18 to 30 (*M* = 21.71, *SD* = 2.35) and 44 percent of them were female. They were randomly assigned to one of the experimental conditions in a three-cell design: individual target, low entitativity group target, and high entitativity group target.

#### Procedure

Participants were recruited in various public facilities of the university, invited to come to the lab for a study supposedly on learning behavior. The experimenter guided the participant into the lab where the confederate(s) was/were already waiting. The experimenter gave participants a short explanation about the study and then turned his back to the participants to get the consent forms. At that moment, the offender (a confederate) showed him “the finger” behind his back. After a fake random draw, the participant was assigned the teacher’s role and the confederate(s) the students’. The participant was then seated in front of a computer and asked to answer a few questions purportedly connected to the student or the students’ group (depending on the condition, see Independent variable below), seated in another room. After a few filler tasks, the main measures were assessed.

#### Independent variable

We manipulated the target of the punishment through the number of confederates and the way they were dressed. In the *individual* condition, one male confederate was sitting in the lab and committed the wrongdoing. In the group conditions, two male confederates were present and sitting in the lab and a third one came in shortly after the participant (it was mentioned that he quickly went to the bathroom). In the *low entitativity group* condition, the three confederates were dressed casually and acted as strangers towards one another. In the *high entitativity group* condition, the three confederates were dressed with the same field-hockey sweaters and greeted each other as friends. One of the confederates committed the wrongdoing and the other two did not acknowledge it in any way.

#### Manipulation checks

The instructions read that researchers were interested in the teacher’s (i.e., the participant) perceptions of their student(s). The same items as in Experiment 1 measured perceptions of entitativity, except that the target was “this student/students’ group” (α = .82, 1 = *not at all*, 7 = *absolutely*).

#### Dependent variable

Participants indicated the extent to which they thought *the student(s) should be punished in one way or another* (1 = *not at all*, 7 = *absolutely*), what punishment they would consider *fair*, *appropriate* and *justified* (1 = *very mild punishment*, 7 = *very severe punishment*). These four general punishment items were aggregated in a measure of support for punishment (α = .92).

### Results

#### Manipulation checks

The group was perceived as less entitative in the low entitativity condition than in the in high entitativity condition, *t*(39) = -3.06, *p* = .004, indicating that our manipulation was successful.

#### Dependent variable

We performed planned orthogonal contrasts analyses similar to Experiment 1, using regression analyses. We created two orthogonal contrasts testing whether support for punishment was significantly higher in the individual condition as compared to both group conditions (C1: 2, -1, -1), and lower in the low entitativity group condition as compared to the high entitativity group condition (C2: 0, -1, 1). Results showed that the first contrast was not significant, C1: *B* = -.15, CI_95%_ [-.36; .07], β = -.17, *t*(61) = -1.35, *p* = .183, *d* = .07, but the second one was right on the significance threshold, C2: *B* = .38, CI_95%_ [-.001; .752], β = .25, *t*(61) = 2.00, *p* = .050, *d* = .17, indicating that support for punishment was higher in the high entitativity condition as compared to the low entitativity condition. These findings further support the hypothesized role of group entitativity in the extent to which people support collective punishment.

Further analyses indicated that support for punishment in the individual condition was higher than in the low entitative group condition, *B* = -.81, CI_95%_ [-1.56; -.06], β = .31, *t*(61) = 2.16, *p* = .035, but did not differ from the support for punishment observed in the high entitative group condition, *B* = -.06, CI_95%_ [-.80; .68], β = .02, *t*(61) = -.16, *p* = .873, explaining why the first contrast turned out non-significant.

### Discussion

Results were consistent with the hypotheses and with results of Experiment 1: Support for punishment was higher in the high entitative group condition as compared to the low entitative group. The punishment of the high entitative group actually was at the same level as the punishment ascribed to the individual wrongdoer, although this absence of difference should be considered with caution given the small sample size of this experiment. These consistent findings suggest that the same dynamics observed in Experiment 1 operated in Experiment 2 as well. Still, it is worth mentioning that even though participants did observe a confederate performing an offensive act prior to making their punishment judgments (an experimenter made sure all participants did indeed witness the offensive act), no explicit association between the wrongdoing and the punishment was made by the experimenters. It was important to do so, as asking directly about the incident would be likely to increase suspicion among participants that the offensive act was an intended aspect of the study. Our conclusions hinge on the assumption that participants would seize that opportunity to punish the offensive act even in the absence of such an explicit link, also in light of the fact that there were no other offensive acts during the study that would realistically motivate punishment. Furthermore, the present design does not exclude the possibility that participants expected a disapproving reaction of the other group members particularly when they seemed to be close friends with the offender (i.e., the high entitativity condition). In Experiment 3, we addressed all of these concerns: We tested the predicted effect with an explicit link between wrongdoing and collective punishment, like we had done in Experiment 1, and the paradigm did not create any expectations about the other group members’ reaction to the wrongdoing.

## Experiment 3

We aimed to replicate the findings from the Experiments 1 and 2 while correcting for some of their methodological limitations, and testing whether the results can generalize across different situations. As such, we conducted a second online experiment focusing on the critical condition, i.e. the (low and high entitativity) group conditions. We presented participants with a group of students who were working together on an assignment. The wrongdoing consisted of a plagiarism committed by one student in the group. We again measured support for collective punishment and expected it to be higher in the high entitativity group condition than in the low entitativity condition. We also improved our methods by including several attention checks, which are useful in online studies in which some participants are likely to pay little attention, creating noise in the data.

### Method

#### Participants and design

We posted the survey on Amazon’s Mechanical Turk and requested 250 workers, aiming at a final sample of a minimum of 100 per cell (and knowing that many participants on this platform often fail attention checks). A total of 262 participants completed the survey (again, it is common on Amazon’s Mechanical Turk for a few more people than requested to take the survey). We excluded participants who failed one of the attention checks (9 in the low entitativity condition and 24 in the high entitativity condition failed to report the correct group composition; 33 participants failed to report what the wrongdoing was, and 24 failed a question asking them to simply select the second point of a scale; these are not mutually exclusive). The final sample consisted of 209 participants, which, according to a sensitivity power analysis, yields 80% power for a small effect size (*f*^*2*^ = .03). Their mean age was 35.71 (*SD* = 10.84), and they were 49% female. Participants were randomly assigned to one of two conditions in a 2-cell design (low versus high group entitativity). Participants were thanked and debriefed at the end of experiment, and were paid USD 0.80 for their participation.

#### Procedure

Participants read about an alleged professor of modern history who requires his students to work in groups to write an assignment. We manipulated the entitativity of the student group on the first page of the vignette (see below). The second page described the wrongdoing: One of the students, who was in charge of assembling the final copy, found a published article online that treated exactly the same topic as their assignment. He inserted large parts of that article in his own group’s assignment. The students then turned their paper in to the professor with the plagiarized text included in their final document.

#### Independent variable

In the low entitativity condition, the vignette read that “*the members of the group were students who had not found a group yet*, *and gathered together randomly*. *They did not know each other before and were very different in terms of backgrounds*, *coming from different countries”*. In the high entitativity condition, the vignette read that “*The members of this group were all part of the same football team*, *and gathered together out of affinity*. *They had been friends for years and were very much alike in terms of background*, *coming from the same town*”.

#### Manipulation checks

We assessed group entitativity at the end of the survey with 6 items following previous research [32]. On a 7-point scale (1 = *Not at all*, 7 = *Absolutely*), participants indicated to what extent they considered that the students from this groups were likely to: *interact with each other*, *influence each other*, *have shared norms*, *have strong interpersonal bonds*, *share knowledge*, *have common goals* (α = .92).

#### Dependent variable

We measured support for collective punishment adapting an individual punishment measure used in previous research (e.g. [33]). After being reminded that the assignment was a group task and that all students signed the paper they submitted, participants indicated on a 7-point scale to what extent they thought the students from the group should be punished one way or another (1 = *Not at all*, 7 = *Absolutely*), and what punishment they considered as fair, appropriate and justified (1 = *No punishment at all*, 7 = *Extremely severe punishment*; α = .86).

### Results

#### Manipulation checks

In the low entitativity group condition, participants reported lower levels of perceived group entitativity as compared to the high entitativity group condition (*t*(207) = -12.78, *p* < .001). We conclude that our manipulation was successful.

#### Dependent variable

Support for collective punishment was higher in the high entitativity group condition as compared to the low entitativity group condition (*t*(207) = -2.58, *p* = .011; *d* = .18). This finding supports our hypothesis.

### Discussion

This third experiment replicates the findings from Experiments 1 and 2, using a different paradigm with a new type of groups and a different wrongdoing, as well as improved methodology. The successful third replication of the effect of group entitativity on support for collective punishment gives us confidence that this effect is real and not limited to specific wrongdoings, but generalizable to different groups and wrongdoings.

## General discussion

Individual punishment has typically been shown to be strongly dependent on ascriptions of blame or responsibility to the wrongdoer [4,34,35], meaning that in the absence of guilt, the infliction of a punishment is generally evaluated as unfair [36]. Support for such punishments was evidenced in the present experiments: Innocent people were the target of collective punishments. People generally consider such collective punishments as unfair, because they involve the punishment of innocent people whose only association with the wrongdoing is their shared membership with the actual wrongdoer(s). The present research investigates one factor likely to increase support for such punishments, namely the group’s entitativity.

Because higher levels of entitativity increase perceptions of a group’s internal cohesion and of its members’ interchangeability, we reasoned that people should be more willing to inflict collective punishments upon high entitative groups as compared to low entitative groups. We found support for this reasoning across three experiments: After a wrongdoing perpetrated by a (few) group member(s), highly entitative groups were punished more severely than lowly entitative groups. In line with our predictions, we additionally observed the highest support for punishment when the target was a single offender, and the lowest when the target was a single innocent individual. The first and third experiments were vignette studies conducted online, presenting participants with different types of groups and wrongdoings. The second experiment replicated the findings in the lab: Participants were left to infer group entitativity through the confederates’ clothing and behavior, and presented with a staged wrongdoing perpetrated by one of the confederates, allowing for a more spontaneous processing of the situation. This methodological diversity suggests that the findings observed here are not artifacts of one specific research setting, but can generalize across situations.

The present work is different from earlier studies on the punishment of groups in several regards. First of all, earlier work on the effect of group entitativity had focused on different punishment types, such as vicarious punishment [21,22], group punishment [23], and retaliatory collective punishment [24,25]. Our focus here was on collective punishment, defined as the infliction of a negative sanction from an external agent to an entire group, including innocent individuals, for the misdeed of a few group members [9], and the present research is hence the first one to show how it is affected by group entitativity both in several vignette studies and in a lab study with a real offense. In addition, respondents in our experiments were not the victims of the original wrongdoing in any way (directly or vicariously), but they still showed willingness to inflict a punishment. Although this is an established finding at the individual level, very few studies have shown it to appear at the collective level.

Moreover, the research described in this paper is the first to compare collective punishment with individual punishment. Consistent with our expectations, it appears that people seem more comfortable with the idea of punishing an individual wrongdoer than an entire group comprising a (few) wrongdoer(s). However, this difference was reduced (Experiment 1) and even non-significant (Experiment 2) when the group was highly entitative, suggesting that highly entitative groups might be treated in an almost similar way to single individuals when it comes to retributive justice judgments. This finding is consistent with research showing that highly entitative groups elicit the same type of information processing as individual targets [14,37]. Could it be that when considering the punishment of highly entitative groups, the same underlying mechanisms as in individual punishment are activated? Considering that the present work only provides limited evidence for such reasoning, future research should examine whether this is the case.

Another interesting question that emerges from the comparison between individual and collective punishment is how they are related to one another. The present research only assessed individual punishment and collective punishment in between-subjects designs, but not *both* at the same time in a within-subjects design. One can speculate on how individual and collective punishment relate to each other based on research related to mind perceptions in individuals and groups. On the one hand, research has shown that perceived entitativity increases the extent to which people attribute a mind to the group, and decreases the extent to which they attribute a mind to each individual group member [18], indicating that people make a trade-off in their mind attributions to groups versus group members as a function of group entitativity. On the other hand, mind attributions increase perceptions of responsibility as well as punishment severity [38]. One might hence expect higher levels of entitativity to produce such a trade-off in punishment judgments as well. Collective punishment would then be *negatively* related to the level of punishment that people assign to the individual wrongdoer within the group. This is a promising question for future research to explore.

As underlined earlier, collective punishments are generally perceived as illegitimate because they violate the basic justice principle according to which punishments should be ascribed in proportion to an offender’s responsibility [34]. Because of this apparent contradiction, the role of responsibility perceptions in support for collective punishments should be particularly interesting to investigate. Previous research has shown that perceptions of collective responsibility are indeed positively correlated with support for collective punishment [9,21], but it appears that this link is not as straightforward as it is at the individual level: For example, perceptions of collective responsibility were less predictive of support for collective punishment when the offender group is less valued, such as a nondemocratic group [10]. This suggests that in order to fully understand how perceptions of responsibility shape support for collective punishments, one needs to consider additional group-level factors that moderate this link.

Group entitativity could be another one of these moderators. Indeed, entitativity perceptions are closely related to homogeneity perceptions [39,40], and higher levels of homogeneity have been shown to reduce the extent to which collective responsibility predicts support for collective punishment [41]. Hence, at high levels of entitativity or homogeneity, people seem to rely less on responsibility perceptions in their punishment judgments. Considering that people tend to view outgroups as more homogeneous than ingroups [42,43], this suggest that people are likely to be more prone to support collective punishments of outgroups regardless of their perceived responsibility, because they are perceived as more homogeneous and entitative. This might be one of the mechanisms triggering increased intergroup conflicts.

A limitation of the present work is the measure of punishment, which is self-reported support for punishment rather than actual punitive behavior. Hence, our understanding of these dynamics would greatly benefit from a behavioral measure of punishment. Although many studies examined punitive behavior in social dilemmas [44] and in mock jury simulations [45], no experimental studies have yet considered a behavioral indicator of collective punishment. Future research should develop a paradigm allowing for a behavioral measure in a setting comparing individual with collective punishment.

Another limitation of the present studies is that our manipulation of group entitativity, in addition to referring to the group members being homogeneous, similar and friends, also partially relied on them being members of the same sports team. We made this choice in order to maximize the perception of high entitativity and based on previous research [e.g. 13]. It is therefore possible that participants in our studies are prejudiced against sport-related youth and that this prejudice could be partially driving the effects. We are not aware of any research showing the existence of a generalized prejudice against people in sports teams, and we would argue that if any such prejudice exists, it is likely itself grounded in high entitativity perceptions. Nonetheless, future research should use different manipulations of entitativity which do not refer to sports team memberships in order to exclude this possibility.

To conclude, our findings shed some light on the reasons why collective punishments are so common in everyday life. Highly entitative groups, such as soccer supporters, are more likely to be the target of collective punishments after a wrongdoing perpetrated by a subset of the entire group. It is likely that the fact that the soccer club Ajax had to collectively suffer for the misbehavior of a few of its supporters was perceived as more legitimate because it is perceived as a very entitative group. We conclude that perceived group entitativity is a potent factor to explain why we sometimes punish the innocent.

## Supporting information

S1 File Mediation analyses for other variables measured in all experiments. (DOC)
