# Supplementary material for: Why we sometimes punish the innocent: The role of group entitativity in collective punishment
Source: PLoS One. 2018 May 3;13(5):e0196852. doi: 10.1371/journal.pone.0196852 (PMC5933726; doi:10.1371/journal.pone.0196852)
Supplement: S1 File — (DOC) [file pone.0196852.s001.doc]

**Online Supplementary Materials**

Why we sometimes punish the innocent: The role of group entitativity in collective punishment

# Mediation analyses

We also measured a range of other variables that we speculated could be potential mediators of the effects evidenced in our article. Whereas we believe that reporting these results in the main article would distract from our main findings, for the purpose of full disclose we decided to present these measures and the exploratory analyses performed in the present online supplementary materials.

## Experiment 1

We additionally included a series of measures in the survey with the goal of examining potential mediation effects in a more exploratory fashion. The literature has shown many factors to be related to punishment. For example, ascriptions of punishment and wrongdoing legitimacy perceptions are related concepts. Indeed, punishments reflect the extent to which wrongdoings are considered as violations of consensual values [1,2]. Previous research has shown that the effect of group political structure on support for collective punishment was mediated by perceptions of group value [3]. Also, it is well documented that retributive responses are strongly influenced by affective reactions [1,4]. Finally, previous research also suggests that victim blaming could be related to the willingness to punish [5]. Consequently, we measured wrongdoing legitimacy perceptions, target value ascriptions, affective reactions and victim blaming tendencies, and examine how they relate to support for punishment and to our manipulation, and whether they are capable of explaining the effects we describe in the main paper.

**Measures.** All items were measured on 7-points scales from 1 = *Not at all, to* 7 = *Absolutely*. We measured *wrongdoing legitimacy* by having participants indicate the extent to which they considered the teenagers’ behavior as: severe (reversed score), violent (reversed score), normal, unacceptable (reversed score), understandable, excusable, unfair (reversed score) and legitimate (α= .95). To measure *target value*, participants indicated to what extent they thought the teenager or the teenager’s group was: moral, violent (reversed score), fair, dishonest (reversed score), respectful, good people/a good person (depending on the condition), bad (reversed score) (α= .95). We measured *affect* by asking participants the extent to which they were feeling angry, irritated, satisfied, outraged, mad, pleased, content, perturbed, happy, and frustrated on a 7-points scale (1 = *Not at all,* 7 = *Absolutely*). Factor analyses revealed two factors. The first factor encompassed all positive emotions and the second one the negative ones. Accordingly, we computed a score of *positive affect* including satisfied, pleased, content and happy (α= .96), and a score of *negative affect* including all remaining items (α= .95). Finally, we measured v*ictim blaming.* Participants were asked, concerning the inhabitants of the village and their actions, how much they considered this event to be their fault, how much they blamed them for what happened, to what extent were they responsible for what happened, and to what extent were they the cause of this event (α= .97).

**Results.** We first examined the predictive power of each of the potential mediators on support for punishment. We regressed support for punishment on target value, wrongdoing legitimacy, positive affect, negative affect and victim blaming. Results indicated that punishment was lower as a function of perceptions of target value, *B* = -.57, β = -.53, *t*(197) = -6.78, *p* < .001, and wrongdoing legitimacy, *B* = -.30, β = -.29, *t*(197) = -3.43, *p* = .001, but higher as a function of positive affect, *B* = .22, β = .18, *t*(195) = 3.29, *p* = .002. No other effects were significant (*p*s > .242). Accordingly, target value, wrongdoing legitimacy and positive affect were identified as possible mediators of the effect of condition on support for punishment.

Accordingly, we proceeded to the analysis of the indirect effects of the variables identified to have an impact on support for punishment. Using the PROCESS macro by Preacher and Hayes [6], we performed a multiple mediators mediation analysis including support for punishment as the dependent variable, Contrast 3 as the independent variable (C3: 0, 0, -1, 1), and all other contrasts as covariates (C1: -3, 1, 1, 1, and C2: 0, 2, -1, -1) and the following parallel mediators: target value, wrongdoing legitimacy, and positive affect. We first report the results of the models tested on each individual mediator, and then the mediation results themselves.

Perceptions of *target value* differed as a function of conditions: The control group was more valued than the three wrongdoers groups, C1: *B* = -.70, CI_95%_ [-.80; -.62], *t*(199) = -15.15, *p* < .001. The individual wrongdoer was less valued than the groups’ wrongdoers, C2: *B* = -.27, CI_95%_ [-.41; -.14], *t*(199) = -4.04, *p* =.001. Finally, the highly entitative group was less valued than the lowly entitative one, C3: *B* = -.34, CI_95%_ [-.56; -.11], *t*(199) = -2.99, *p* = .003. *Wrongdoing legitimacy* was higher in the control condition as compared to the wrongdoing conditions, C1: *B* = -.91, CI_95%_ [-.98; -.84], *t*(199) = -24.84, *p* < .001. No other effects were significant (*p*s > .525). Participants also reported higher levels of *positive affect* in the control condition as compared to the wrongdoing conditions, C1: *B* = -.41, CI_95%_ [-.52; -.31], *t*(199) = -7.69, *p* < .001. No other effects were significant (*p*s > .324).

Once the mediators were entered in the model, the direct effects of Contrast 1 and Contrast 3 were no longer significant: C1: *B* = .16, CI_95%_ [-.04; .36], *t*(196) = 1.59, *p* = .114; C3: *B* = .08, CI_95%_ [-.16; .32], *t*(196) = .658, *p* = .511. Contrast 2 remained significant, C2: *B* = .19, CI_95%_ [.05; .35], *t*(196) = 2.55, *p* = .012, indicating that the individual wrongdoer was still punished more harshly than the group wrongdoers after controlling for the effects of target value, wrongdoing legitimacy, and positive affect. Punishment was also lower as a function of the ascribed target value, *B* = -.50, CI_95%_ [-.68; -.33], *t*(196) = 5.74, *p* < .001, and as a function of perceptions of wrongdoing legitimacy, *B* = -.25, CI_95%_ [-.47; .03], *t*(196) = -2.20, *p* = .029, but higher as a function of positive affect, *B* = .21, CI_95%_ [.07; .34], *t*(196) = 3.01, *p* = .003.

Consistent with these findings, the indirect effect through target value was significant, *B* = .17, *SE* = .07, CI_95%_ [.06; .32], *p* = .009. However, the indirect effects through legitimacy was non-significant, CI_95%_ [-.02; .08], as was the indirect through positive affect, CI_95%_ [-.01; .08]. We hence conclude that the differences in support for punishment between the control and the wrongdoing conditions, as well as between the low and high entitative groups are mediated by perceptions of target value in this experiment. However, it appears that the difference in punishment between the individual conditions and the group conditions depends on other factors.

## Experiment 2

Preliminary and exploratory findings from Experiment 1 pointed towards the mediating role of perceptions of target value. Accordingly, we examined the mediating role of this variable again in Experiment 2. We additionally included wrongdoing legitimacy perceptions again, considering that it had quite a strong effect of support for punishment and that its indirect effect just fell short of significance in Experiment 1.

**Measures.** All items were measured on 7-points scales from 1 = *Not at all, to* 7 = *Absolutely*. *Wrongdoing legitimacy* was measured by having participants indicate the extent to which they thought this behavior was: *acceptable*, *understandable*, *legitimate* and *fair* (α= .87). *Target value* was measured by having participants indicate, regarding the target, to what extent they: *liked them, thought they were good people, thought they were moral people, thought they were respectful people, did they respect them, and did it make a positive impression on them* (α= .91).

**Results.** We first regressed support for punishment on target value and wrongdoing legitimacy, in order to assess whether they could be suitable mediators. Results showed no significant effects (*p*s > .137). We hence conclude that target value and wrongdoing legitimacy cannot be mediators of the effect of group entitativity on support for punishment. In spite of that, analyses using PROCESS indicated that once the mediators were entered in the model the effect of Contrast 2 was only marginally significant, *B* = .37, CI_95%_ [-.015; .76], *t*(57) = 1.92, *p* = .059. However, none of the indirect effects through the mediators were significant: target value CI_95%_ [-.09; .14], and wrongdoing legitimacy CI_95%_ [-.07; .06]. These findings confirm that neither target value nor wrongdoing legitimacy are mediators of the effect of group entitativity on support for punishment.

## Discussion

Results across both experiments show inconsistent results. Therefore we decided not to include these measures in the main document, as it is unclear for us what to conclude from them. Future research should investigate more closely the effects of perceptions of target value, as Experiment 1 reveals suggestive, but only preliminary, evidence for their existence. For the present purposes, we restrict our conclusions to the effects of group entitativity on collective punishment, for which two independent studies with different operationalizations—one Internet experiment and one lab study including confederates—revealed highly consistent results.

## Experiment 3

In Experiment 3, we wanted to explore what could drive such willingness to inflict punishments upon innocent group members. Motives underlying *individual* punishment include retribution, deterrence, incapacitation, rehabilitation and victim compensation [2]. To our knowledge, no research has investigated whether and how these punishment goals shape support for *collective* punishments. Firstly, support for collective punishments could be guided by a just deserts rationale. On the one hand, it could be that the desire to see the offender punished is so strong that it needs to be satisfied no matter what: The fact that innocent people are punished in the process would be a simple “collateral damage” that people are willing to accept. If this would be the case though, one would not expect groups’ characteristics, such as its entitativity, to affect punishment judgments. The findings from our paper make such an interpretation unlikely. On the other hand, the retributive motives triggered by the offense might be displaced from the actual offender to the rest of his group. This process is likely to be facilitated by a higher group entitativity: The urge to punish would easily propagate among all members of highly entitative groups because they are perceived as similar and interchangeable—they are all the same, hence, they are all guilty. Indeed, this line of reasoning converges with findings showing that punitive judgments can be influenced by other group-based factors that create impressions of similarity, such as stereotypes [7–10]. The present findings hence would be consistent with such an understanding. Retributive motives are related to emotional arousal such as the experience of moral outrage. If the retributive motive is spread from the offender to his group and that this spread is facilitated by higher levels of group entitativity, then the feeling of moral outrage should also spread more easily as a function of group entitativity. We hence measured feelings of outrage and explored whether it mediates the effect of group entitativity on support for collective punishment.

Secondly, support for collective punishments could be guided by deterrence motives. Indeed, an argument that is often made in favor of collective punishments is that they would be an effective way to prevent future offenses by increasing internal regulation [11,12]. One can imagine that this expectation of internal control could be higher in highly entitative groups, as members of such groups are likely to be in a better position to apply such control over one another. If that is true, then perceptions of the importance of preventing future offenses should be higher in highly entitative groups as compared to lowly entitative groups, and should mediate the effect of group entitativity on support for collective punishment.

If such internal control is expected from members of groups, then people should perceive group members as causally or morally *responsible to prevent offenses* by fellow group members. Such responsibility perceptions are likely to be increased as s function of group entitativity. Furthermore, previous research has shown that perceptions of collective responsibility for wrongdoings by a few group members increase support for collective punishment [13]. In addition, higher levels of collective entitativity have been shown to lead to higher perceptions of collective responsibility [14]. Therefore, we could expect perceived causal or moral responsibility to prevent wrongdoings to lead to similar effects and to be higher among highly entitative groups as compared to lowly entitative groups. As a result, we measured *causal* and *moral responsibility* *to prevent the wrongdoing* and expected those perceptions to mediate the effect of group entitativity on support for collective punishment.

**Measures.** We measured all construct with single items: “*To what extent are you outraged at the entire group for this plagiarism?*”; “*To what extent do you believe that it is important to prevent this behavior from happening in the future?*”; “*To what extent do you think that the other group members could have prevented the plagiarism? (they had the opportunity to do so)*”; “*To what extent do you think the other group members should have prevented the plagiarism? (they had the responsibility to do so)*”.

**Results.** All variables correlated positively and significantly with support for punishment: moral outrage (*r*(262) = .57), prevention importance (*r*(262) = .11), causal responsibility (*r*(262) = .39), and moral responsibility (*r*(262) = .35). We then regressed all variables on group entitativity. Results showed no significant effects (*p*s > .127). This suggests that these variables cannot be mediators of the effect of group entitativity on support for punishment. Analyses using PROCESS confirmed that the effect of group entitativity remained positive and significant when including all variables as mediators of that effect. Furthermore, none of the indirect effects were significant.

**Discussion**

Because of these inconclusive findings, we decided to exclude these measures and analyses from the main paper and to focus on the effect of group entitativity on support for third party collective punishment, for which the evidence is consistently supportive.

# Regression analyses controlling for gender and age

## Experiment 1

**Dependent variable.** We performed planned orthogonal contrasts analyses using regressions analyses and controlling for gender and age. We created three orthogonal contrasts testing whether support for punishment was significantly lower in the control condition as compared all three conditions where an offense was committed (C1: -3, 1, 1, 1), higher in the individual condition as compared to both group conditions (C2: 0, 2, -1, -1), and lower in the low entitativity group condition as compared to the high entitativity group condition (C3: 0, 0, -1, 1), the latter being the critical test of our hypothesis. Results show that all three contrasts were significant. The target was punished less severely in the control condition as compared to all other conditions, C1: *B* = .65, CI_95%_ [.54; .77], β = .62, *t*(197) = 11.46, *p* < .001. Support for the individual punishment was also higher than support for the group conditions, C2: *B* = .36, CI_95%_ [.19; .52], β = .22, *t*(197) = 4.26, *p* < .001. Finally, support for collective punishment was higher when the group’s entitativity was high rather than low, C3: *B* = .30, CI_95%_ [.02; .57], β = .12, *t*(197) = 2.15, *p* = .033. This latter finding supports our hypothesis. Neither age nor gender had any significant effect (*p*s > .76).

## Experiment 2

**Dependent variable.** We performed planned orthogonal contrasts analyses similar to Experiment 1, using regressions analyses and controlling for gender and age. We created two orthogonal contrasts testing whether support for punishment was significantly higher in the individual condition as compared to both group conditions (C1: 2, -1, -1), and lower in the low entitativity group condition as compared to the high entitativity group condition (C2: 0, -1, 1). Results showed that the first contrast was not significant, C1: *B* = -.14, CI_95%_ [-.36; .07], β = -.16, *t*(57) = -1.32, *p* = .192, but the second one was, C2: *B* = .39, CI_95%_ [.02; .77], β = .26, *t*(57) = 2.09, *p* = .042, indicating that support for punishment was higher in the high entitativity condition as compared to the low entitativity condition. These findings further support the hypothesized role of group entitativity in the extent to which people support collective punishment.

Further analyses indicated that support for punishment in the individual condition was higher than in the low entitative group condition, *B* = -.82, CI_95%_ [-1.57; -.07], β = .31, *t*(57) = 2.18, *p* = .033, but did not differ from the support for punishment observed in the high entitative group condition, *B* = -.03, CI_95%_ [-.78; .71], β = .01, *t*(57) = .09, *p* = .927, explaining why the first contrast turned out non-significant. Neither age nor gender had any significant effect (*p*’s > .13).

## Experiment 3

**Dependent variable.** We performed an ANOVA controlling for gender and age. Support for collective punishment was higher in the high entitativity group condition as compared to the low entitativity group condition (*F*(207) = 5.59, *p* = .019). The effect of gender was non-significant (*p* = .506), but age had a significant negative effect on support for collective punishment (*F*(207) = 8.07, *p* = .005). Because this effect doesn’t change our main finding and isn’t consistent with previous experiments, we do not investigate any further.

# References

1 Darley, J.M. and Pittman, T.S. (2003) The Psychology of Compensatory and Retributive Justice. *Personal. Soc. Psychol. Rev.* 7, 324–336

2 Vidmar, N. and Miller, D.T. (1980) Socialpsychological Processes Underlying Attitudes toward Legal Punishment. *Law Soc. Rev.* 14, 565–602

3 Falomir-Pichastor, J.M. *et al.* (2007) Perceived Legitimacy of Collective Punishment as a Function of Democratic versus Non-Democratic Group Structure. *Group Process. Intergroup Relat.* 10, 565–579

4 Wenzel, M. and Okimoto, T.G. (2016) Retributive Justice. DOI: 10.1007/978-1-4939-3216-0

5 Hafer, C.L. and Begue, L. (2005) Experimental Research on Just-World Theory: Problems, Developments, and Future Challenges. *Psychol. Bull.* 131, 128–167

6 Preacher, K.J. and Hayes, A.F. (2008) Asymptotic and resampling strategies for assessing and comparing indirect effects in multiple mediator models. *Behav. Res. Methods* 40, 879–891

7 Bodenhausen, G.V. and Wyer, R.S. (1985) Effects of stereotypes on decision making and information-processing strategies. *J. Pers. Soc. Psychol.* 48, 267–282

8 Van Prooijen, J.W. *et al.* (2014) Power and retributive justice: How trait information influences the fairness of punishment among power holders. *J. Exp. Soc. Psychol.* 50, 190–201

9 Sommers, S.R. *et al.* (2000) Race in the Courtroom : Perceptions of Guilt and Dispositional Attributions. *Pers. Soc. Psychol. Bull.* 26, 1367–1379

10 Heckathorn, D.D. (1990) Collective sanctions and compliance norms: A formal theory of group-mediated social control. *Am. Sociol. Rev.* 55, 366–384

11 Heckathorn, D.D. (1988) Collective Sanctions and the Creation of Prisoner’ s Dilemma. *Am. J. Sociol.* 94, 535–562

12 Lipnowski, I. (1993) Partial Rehabilitation of the Principle of Collective Punishment, A. *Can. J. Law Soc.* 8, 121

13 Pereira, A. *et al.* (2015) Collective punishment depends on collective responsibility and political organization of the target group ☆. *J. Exp. Soc. Psychol.* 56, 4–17

14 Lickel, B. *et al.* (2003) A case of collective responsibility: who else was to blame for the Columbine high school shootings? *Pers. Soc. Psychol. Bull.* 29, 194–204
